# Two Defensive Lines in Juvenile Leaf Beetles; Esters of 3-nitropropionic Acid in the Hemolymph and Aposematic Warning

**DOI:** 10.1007/s10886-016-0684-0

**Published:** 2016-03-31

**Authors:** Gerhard Pauls, Tobias Becker, Peter Rahfeld, Rene R. Gretscher, Christian Paetz, Jacques Pasteels, Stephan H. von Reuss, Antje Burse, Wilhelm Boland

**Affiliations:** Department of Bioorganic Chemistry, Max Planck Institute for Chemical Ecology, Jena, Germany; Biosynthesis and Nuclear Magnetic Resonance Group, Max Planck Institute for Chemical Ecology, Jena, Germany; Department of Biology, Université Libre de Bruxelles, Brussels, Belgium

**Keywords:** Chrysomelidae, Chemical defense, 3-nitropropionate esters, Isoxazolin-5-one glucoside, Aposematic warning

## Abstract

Juveniles of the leaf beetles in subtribe Chrysomelina have efficient defense strategies against predators. When disturbed, they transiently expose volatile deterrents in large droplets from nine pairs of defensive glands on their back. Here, we report on an additional line of defense consisting of the non-volatile isoxazolin-5-one glucoside and its 3-nitropropanoyl ester in the larval hemolymph. Because isoxazolin-5-one derivatives were not detectable in related leaf beetle taxa, they serve as a diagnostic marker for the Chrysomelina subtribe. Conjugation of isotopically labelled 3-nitropropionic acid to isoxazolin-5-one glucoside *in vivo* demonstrates its function as a carrier for the 3-nitropropanoyl esters. The previous identification of characteristic glucosides as precursors of the volatile deterrents underlines the general importance of glucosides for sequestration from food plants, and the subsequent transport in the hemolymph to the defense system. The combination of repellent volatiles with non-volatile toxic compounds in the hemolymph has the potential to create synergistic effects since the odorant stimulus may help predators learn to avoid some foods. The combination of the two defense lines has the advantage, that the hemolymph toxins provide reliable and durable protection, while the repellents may vary after a host plant change.

**Electronic supplementary material:**

The online version of this article (doi:10.1007/s10886-016-0684-0) contains supplementary material, which is available to authorized users.

## Introduction

Toxins are the most effective players on our planet when it comes to the manifold interactions in trophic networks. Reflecting successful relationships with their hosts, leaf beetles (family Chrysomelidae) of the taxon Chrysomelina have ingenious strategies that disarm plant toxins and, at the same time, produce a chemical defense against natural enemies. This chemical defense not only protects all developmental stages from larvae to adults, but also changes its composition during the life history of Chrysomelina beetles.

Adult beetles store and release defensive secretions from pronotal and elytral exocrine glands upon disturbance (Deroe and Pasteels 1982; Pasteels *et al.*^1989^). The major components are isoxazolin-5-one glucosides esterified with up to three 3-nitropropionic acid (3-NPA) moieties (Pasteels *et al.*^2003^; Sugeno and Matsuda 2002). As these compounds are originally not present in any of the beetle host plants, they are postulated to be synthesised by the insects themselves *via* pathways that remain to be explored (Randoux *et al.*^1991^).

While the mode of action of the isoxazolinone moiety is unknown, 3-NPA is a naturally occurring neurotoxin that, when ingested, causes poisoning in both humans and domestic livestock by irreversibly inhibiting the mitochondrial succinate dehydrogenase (E.C. 1.3.5.1), a key enzyme of the citric acid cycle (Anderson *et al.*^2005^; Beal *et al.*^1993^; Huang *et al.*^2006^). 3-Nitropropionic acid and its glucose esters have been identified in many members of the legume plant family (Fabaceae) and in certain fungi as a defense against herbivores (Chomcheon *et al.*^2005^; Francis *et al.*^2013^; Parry *et al.*^2011^). Some insect herbivores can detoxify 3-NPA (Johnson *et al.*^2001^; Majak *et al.*^1998^; Novoselov *et al.*^2015^). To date, Chrysomelina leaf beetles are the only insects in which 3-NPA and its derivatives have been described as allomones. The amounts of 3-NPA that predators of leaf beetles, for example ants or birds can tolerate before avoiding further consumption, remain to be determined.

**Fig. 1:**
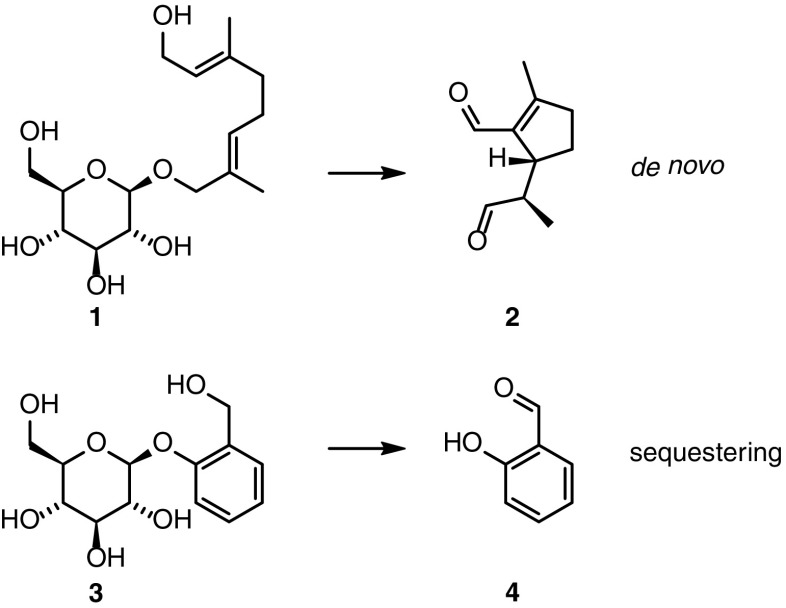
Compounds implicated in the volatile chemical defense of the Chrysomelina larvae. 8-hydroxygeraniol glucoside (**1**) and salicin (**3**) are precursors of chryomelidial (**2**) and salicylaldehyde (**4**) in *Phaedon cochleariae* and *Chrysomela populi*, respectively

Unlike adults, juveniles rely on volatile repellents whose molecular structure is entirely different. By displaying droplets of defensive secretions from nine pairs of everting glandular reservoirs located on their backs, the larvae have an extraordinary defense mechanism unparalleled in the insect world. The defensive chemicals in the Chrysomelina larval exudates are composed of four compound classes (Fig. 1): iridoids (cyclopentanoid monoterpenoids, *e.g.*, chrysomelidial **2**), aldehydes (salicylaldehyde **4** and benzaldehyde), esters (*e.g.*, phenethyl esters), and the naphtoquinone juglone (Hilker and Schulz 1994; Laurent *et al.*^2005^; Matsuda and Sugawara 1980; Pasteels *et al.*^1986^). Phylogenetic analysis of Chrysomelina species revealed that the composition of their secretions reflects a step-wise scenario of host-plant adaptation (Termonia *et al.*^2001^).

The plant-independent biosynthesis of iridoids predated the sequestration of salicin, a plant-derived precursor from Salicaceae, used to produce the repellent salicylaldehyde (Kuhn *et al.*^2004^; Pasteels *et al.*1983b). Later in Chrysomelina beetle evolution, a sequestering Chrysomelina lineage -- namely, the *interrupta* group -- escaped the constraints of their host plant (willow) by shifting to birch. Due to the different secondary metabolites present in the two hosts, this shift resulted in modified larval exudates produced from the sequestered precursors. For example, the willow-feeding population of the species, *Chrysomela lapponica,* produces predominantly salicylaldehyde from sequestered salicin, whereas the birch-feeding population is able to take up a wide variety of glucosidically bound leaf alcohols. These leaf alcohols are further esterified with butyric acid, resulting in a cocktail of at least 60 esters in the defensive exudate (Geiselhardt *et al.*^2015^; Termonia *et al.*^2001^). Regardless of the different composition and origin of the defensive metabolites in the secretions of Chrysomelina larvae (*de novo vs.* sequestration), the synthesis of all Chrysomelina allomones includes glucoside intermediates (Discher *et al.*^2009^).

Owing to their defensive volatiles, the larvae are protected against microbial infestation (Gross *et al.*^1998^, ^2002^, ^2008^; Gross and Schmidtberg 2009; Gross *et al.*^2008^), generalist arthropod predators (Blum *et al.*^1978^; Hilker and Schulz 1994; Palokangas and Neuvonen 1992; Pasteels *et al.*1983a, b), and insectivorous birds (Topp 1997). These nonspecific volatile irritants, however, act as repellents rather than as toxins that target specific physiological processes (Pasteels *et al.*1983a), and their ecological significance has to date remained poorly understood. As toxicity is often associated with warning signals such as colors, sound, taste, or odors (Pasteels *et al.*1983a), we hypothesized that the volatile irritants also may be linked with toxins not yet identified in Chrysomelina larvae. Since the hemolymph provides the storage site for toxins in a wide range of insect species (Laurent *et al.*^2005^; Opitz and Muller 2009), we analyzed the inventory of secondary metabolites in the hemolymph of juvenile chrysomelids by LC-MS and NMR.

Here, we report on the identification of isoxazolin-5-one glucoside and its 6-nitropropanoate in the larval hemolymph of all tested Chrysomelina species. Previously, these compounds had been attributed exclusively to the adults. However, this finding leads to the conclusion that Chrysomelina species are protected by isoxazolinone glucosides and their 3-NPA esters throughout the beetle life history. Hence, in addition to the defensive odor released from their dorsal glands, the larvae possess toxins in their hemolymph. This association may contribute synergistically to protection against an array of vertebrate and invertebrate enemies. Further, we detected glucoside precursors for the volatile secretions in the larval hemolymph, a discovery that underlines the importance of sugar derivatives as carriers for controlled translocation processes and for preventing the insects from self-poisoning.

## Methods and Materials

### Insect Rearing

*Chrysomela populi* (L.) was collected near Dornburg, Germany on *Populus maximowiczii* × *Populus nigra*. Beetles were propagated using a cycle of 16 h L and 8 h D at 18 ± 2 °C in light and 13 ± 2 °C in darkness. *Phaedon cochleariae* (F.) was collected from Brassicaceae close to the city of Bayreuth (Germany) and kept as a continuous culture in the laboratory (Discher *et al.*^2009^). Larvae were reared on *Brassica rapa* subsp. *pekinensis* “Cantonner Witkrop” (Quedlinburger Saatgut, Quedlinburg, Germany) in a Snijder chamber (Snijders Scientific, Tilburg, Netherlands) in a cycle of 16 h L / 8 h D and 13 °C/11 °C ± 1 °C. The low temperature (13 °C) was necessary to reduce fungal growth on the food plant. Willow feeding *C. lapponica* (L.) were collected in the Altai Mountains in East Kazakhstan, near Katon-Karagai in the Katon-Karagaisky State National Nature Park (2100 m altitude). Birch-feeding *C. lapponica* was collected from *Betula rotundifolia* in the Altai Mountains in East Kazakhstan, close to Uryl, near the Burkhat Pass in the Katon-Karagaisky State National Nature Park (2130 m altitude). All other species were collected in the field, see Table S1 for details.

### Preparation of Samples from the Hemolymph, Frass, and Whole Larvae Extracts

Hemolymph samples were collected as described previously (Bodemann *et al.*^2012^) in capillaries that were sealed immediately after collection and stored at −20 °C until use. Hemolymph weight was determined by measuring the weight of a filled capillary minus its dry empty weight (Mettler-Toledo XS 205, Greifensee, Switzerland). For LC-MS measurements, the hemolymph was diluted with 50 % aqueous MeOH in a ratio of 1 μl hemolymph per 100 μl solvent. Frass samples of *P. cochleariae* (2.5 mg), *C. populi* (13 mg), and *C. lapponica* (5 mg) were extracted with water and analyzed by LC-MS.

For crude extracts prepared from complete larvae, each larva was weighed individually using an ultra-microbalance (XS205; *d* = 0.01 mg; Mettler-Toledo, Greifensee, Switzerland). Individual larvae were frozen separately in liquid N_2_ and macerated in 500 μl MeCN using a Geno grinder. After centrifugation (10,621 rpm, 10 min, room temperature), the supernatant was subject to LC-MS analysis.

### Analysis of Glucosides by LC–MS

Analyses were carried out using an Agilent HP1100 HPLC system equipped with an RP-C18 column, LiChroCART (250 × 4 mm, 5 μm;Merck KGaA, 64271, Darmstadt, Germany) connected to a Finnigan LTQ (Thermo Electron Corp, Dreieich, Germany) operated in the APCI mode (vaporizer temperature: 500 °C, capillary temperature 300 °C). Standard compounds for identification were either purchased (Sigma-Aldrich (St. Louis, MO, USA) or synthesised. Isoxazolin-5-one glucoside and its esters were synthesised according to previously described protocols (Becker *et al.*^2013^, ^2015^).

Samples were analyzed by injection (5 μl) and by the application of a gradient elution. The following protocol was used: 100 % solvent A (H_2_O + 0.1 % HCOOH) and 0 % solvent B (MeCN + 0.1 % HCOOH), linear gradient to 60 % solvent B in 35 min. Extract samples of whole larvae were analyzed by injecting a 5 μl sample and using an isocratic elution with 35 % solvent B (*v/v*) in H_2_O +0.1 % HCOOH. For identification and quantification, the formic acid adducts [M+HCOOH-H]^−^ were used (*m/z* 292 for 2-(β-D-glucopyranosyl)-3-isoxazolin-5-one (**5)**, *m/z* 393 for 2-[6′-(3″-nitropropanoyl)-β-D-glucopyranosyl]-3-isoxazolin-5-one (**6)**, *m/z* 331for salicin (**3)**, and *m/z* 377 for 8-hydroxygeraniol-8-O-*β*-D-glucoside (**1)**.

### Analysis of Crude Larval Hemolymph by NMR

Hemolymph from 20 larvae of *P. cochleariae* or *C. populi* was collected and taken up in 200 μl CD_3_OD for ^1^H/^2^D-exchange. The solution was concentrated under reduced pressure and dissolved in 500 μl CD_3_OD. One-dimensional ^1^H NMR spectra were recorded on a Bruker AV400 using water suppression (purge). Two-dimensional double quantum-filtered (*dqf*)-COSY spectra with phase cycling were recorded on a Bruker AV400. A total of 32 scans were acquired using a time domain of 8 k in F2 (acquisition time of 1.2 s) and 512 increment in F1. Spectra were zero-filled to 8 k × 4 k prior to Fourier transformation and phasing using the Topspin software (Bruker). Heteronuclear HSQC and HMBC spectra were recorded using Bruker AMX500 with a cryoprobe. Samples were dissolved in 100 μl CD3OD using 2 mm NMR vials. For HSQC spectra, 40 scans were acquired using a time domain of 1 k in F2 and 256 increments in F1. For HMBC spectra, 256 scans were acquired using a time domain of 4 k in F2 and 128 increments in F1. Spectra were zero-filled to 4 k × 2 k prior to Fourier transformation and phasing using the Topspin software (Bruker).

### Statistical Analysis

Linear regressions were used to investigate whether the amount of **5** and **6** changed with the weight of the larvae. In order to achieve homogeneous variances and normality of the residuals, data were square root transformed. Data were analyzed with SigmaPlot 11.0.

### Synthesis of Labelled [1-^13^C, 3-^15^N]-3- Nitropropionic Acid and Injection of Labelled 3-NPA into the Larval Hemolymph

Stable isotope labelled [1-^13^C,3-^15^N]-3-nitropropinoic acid was synthesized according to Baxter *et al.* (1992) by using K^13^CN instead of Na^13^CN, and Na^15^NO_2_ instead of NaNO_2_.

The mass of third-instars (15 days after hatching) was measured on an ultra-microbalance (Mettler-Toledo, Greifensee, Switzerland). Ice-chilled larvae were injected dorso-medianally in the intersegmental membrane behind the pronotum using a pulled glass capillary as a needle connected to a nanoliter-injection pump (WPI, Sarasota, FL, USA), mounted on a three-axis-micromanipulator. For labelling experiments, each larva was injected with 200 ng 3-NPA per mg body weight in 122 nl injection-buffer (Bodemann *et al.*^2012^), representing a sublethal dose determined previously in a pilot experiment. To test larval tolerance to 3-NPA, 10 *P. cochleariae* larvae each were injected with the following concentrations: 100, 200, 300, 400 ng/mg body weight. While 300 ng/mg body weight was tolerated (10 of 10 survived), injection of 400 ng/mg body weight was fatal (10 of 10 died).

## Results

### Identification of Isoxazolinone Glucosides in the Larval Hemolymph of Leaf Beetles

**Fig. 2:**
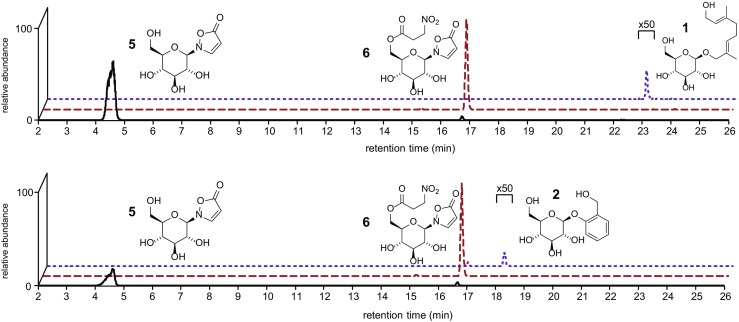
Liquid chromatograms of larval hemolymph of chrysomelina leaf beetles. From top to *bottom*: *Chrysomela populi*, *Phaedon cochleariae*. Traces for formic acid adducts [M+HCOOH]^−^ for glucosides are shown: isoxazoline-5-one-glucoside (*m/z* 292, solid line), isoxazoline-5-one-glucoside 3-NPA ester (*m/z* 393, *dotted* line), salicin (*m/z* 331, dashed line), 8-hydroxygeraniol glucoside (*m/z* 377, *dashed line*)

To detect isoxazolinone glucosides in leaf beetle hemolymph, the following model species were analyzed: *P. cochleariae,* representing the iridoid *de novo* producers; *C. populi* and willow-feeding *C. lapponica,* representing the salicin-sequestering species. The larval hemolymph samples were analyzed by HPLC-MS. APCI ionization was chosen because it is less susceptible to matrix effects (Peters and Remane 2012). Compounds were identified by comparing HPLC-MS chromatograms of the natural samples with the spectra of synthetic standards (Fig. 2, see Fig. S1 and S2 for mass spectra).

To confirm the identity of the presumed hemolymph toxins and to obtain an unbiased assessment of the whole metabolome, we employed NMR spectroscopy. Previous reports demonstrated that ^1^H NMR spectroscopy is well suited to analyze complex metabolome mixtures, including insect hemolymph samples (Lenz *et al.*^2001^; Phalaraksh *et al.*^1999^; Poynton *et al.*^2011^). For our analysis of the crude unfractionated *C. populi* and *P. chochleariae* hemolymph, we used the two-dimensional double quantum-filtered correlation spectroscopy (*dqf*-COSY), which provides outstanding sensitivity and dynamic range along with a wealth of structural information. Analysis of the *dqf*-COSY spectra (Fig. S3) indicated the presence of several free amino acids such as alanine, valine, leucine, isoleucine, threonine, and proline (Table S2), along with characteristic signals corresponding to two dominant *β*-glucosidic components. Their heteroaromatic aglycone moieties were identified as isoxazolinones based on two characteristic AX-spin systems at δ_H_ 8.453 and 5.315 or at δ_H_ 8.433 and 5.344 that both displayed a coupling constant of ^3^*J* = 3.7 Hz. The linkage of the glucose and isoxazolinone moieties was established by complementary HSQC and HMBC correlations from the anomeric hydrogen to the β-carbon. The β-configuration of the glycosidic bond was deduced from the vicinal coupling constant ^*3*^*J*_1’,2’_ = 9.2 Hz for the anomeric hydrogen. Both isoxazolinone glucosides differed in the chemical shifts of the 6′-position, indicating 6′-acylation in one of the two components. This assumption was confirmed by HMBC correlations from the 6′-methylene protons to a carbonyl moiety at δ_C_ 171.5 ppm. Furthermore, this carbonyl group displayed additional HMBC correlations to an A_2_M_2_ spin system at δ_H_ 3.02 δ_C_ 31.7 ppm and δ_H_ 4.71 δ_C_ 70.7 ppm, indicating a 3-nitropropanoate substitution. Comparison of the ^1^H and ^13^C NMR data with data of the authentic standard obtained by synthesis as previously described (Becker *et al.*^2013^, ^2015^) confirmed our structure assignment. Since the α- and β-anomers of compound **5** can be separated easily by LC (RP-C18 column, separation factor *R* = 1.74 (Becker *et al.*^2013^, ^2015^) by using isocratic elution with acetonitrile and water (3:97, v:v), the larval defense compound consists of the pure β-anomer (>99 %).

Both components, isoxazolinone β-glucoside and its 6′-nitropropanoate (Fig. 2, Figs. S1, S2), have been previously described in eggs and adults of the subtribe Chrysomelina (Matsuda and Sugawara 1980; Pasteels *et al.*^1986^), but the presence of these compounds in the larval hemolymph previously was unknown.

### Occurrence of Isoxazolin-5-one Derivatives in Various Chrysomelidae

**Fig. 3:**
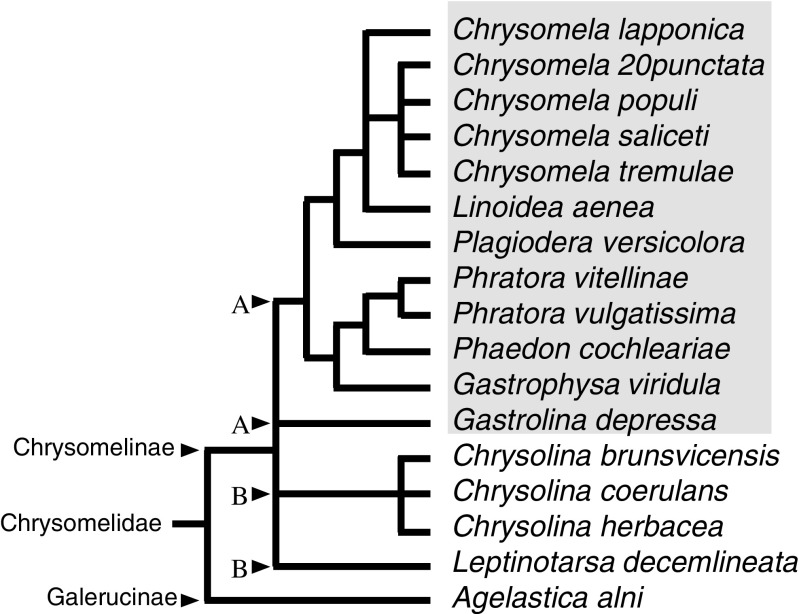
Phylogeny in relation to the presence of isoxazolinone glucoside and its 6-nitropropanoate ester in the hemolymph. The phylogeny is adapted from Termonia and Pasteels (1999), Gomez-Zurita *et al.* (2007), and Daccordi (1994) to represent different Chrysomelidae species in relation to the presence of isoxazolinone glucoside and 6-nitropropanoate ester in the larval haemolymph (marked with *grey box*). The branch points marked with A show the separation into the tribe Chrysomelini (subtribe Chrysomelina) and B into the tribe Chrysolinini (subtribe Doryphorina)

To determine the distribution of isoxazolin-5-one derivatives in the leaf beetle family, the larval hemolymph of Chrysomelina beetles and related subtribes Chrysolinina and Galerucinae were analyzed. In all tested species of Chrysomelina, the isoxazolinone glucoside **5** could be quantified, whereas in the two other subtribes, the glucoside was not detectable (Fig. 3, Table S3). The corresponding nitropropanoyl ester **6** was detectable in the Chrysomelina hemolymph samples of the *de novo* iridoid-producing species *P. cochleariae*, in the salicin-sequestering species *C. populi*, *Chrysomela saliceti, Chrysomela tremulae*, and willow-feeding *C. lapponica* and in the ester-producing *C. lapponica* which feeds on birch (Fig. 3). A limiting factor is the lack of the stability of the 3-NPA esters in the hemolymph, while the isoxazolinone glucoside **5** exhibits as an *N*-glucoside with exceptional stability. Even in acidic media, the compound is stable (Becker *et al.*^2015^). Hence, notwithstanding the production strategy of volatile deterrents in the defensive glands of juvenile Chrysomelina, all tested Chrysolinina species posess a hemolymph-based chemical defense. Leaf beetle taxa other than Chrysomelina do not possess any of the isoxazolin-5-one derivatives.

### Isoxazolin-5-one Derivatives are Produced During Larval Development Endogenously

Considering the high concentrations of compounds **5** and **6** in the larval hemolymph and results reported by (Randoux *et al.*^1991^), who describe the *de novo* production of isoxazolin-5-one derivatives in adult chrysomelids, we asked whether the larvae also are able to synthesize these derivatives endogenously during the juvenile stage. Therefore, we analyzed these compounds in different larval stages in *P. cochleariae* as representatives. Whole larvae extracts were analyzed by LC-MS, and the amount of isoxazolinone glucoside **5** and its 3-NPA ester **6** in nmol/mg plotted *vs.* fresh body weight (ranging from 2 to 15 mg) as shown in Fig. S4. We found that the concentration of compound **5** (in nmol/mg body weight) remained constant (regression analysis, *P* = 0.448) in larvae of different body weight, whereas the concentration of the glucoside ester **6** increased with body weight (*P* < 0.001), indicating that an increasing amount of total isoxazolin-5-one derivatives must be produced during larval development.

In order to find out if the toxin level in the hemolymph can be regulated by excretion *via* the malpighian tubules or hindgut, we analyzed the frass of chrysomeline larvae. However, we could detect neither the glucoside **5** nor the ester **6** in the frass of *P. cochleariae*, *C. populi*, or willow-feeding *C. lapponica* within the detection limits. Both analytes also were not detectable in defensive secretions of the larvae. This suggests that a strongly limited excretion of isoxazolinone derivatives contributes to their accumulation in the hemolymph, which also requires an internal regulation of the ratio of glucoside **5** to ester **6**.

### Free 3-NPA is Conjugated to Isoxazolin-5-one Glucoside

**5** Given the toxicity of free 3-NPA, we tested the metabolic ability of Chrysomelina larvae to accept free 3-NPA as a substrate for the biosynthesis of the ester compound **6**. For this purpose, sublethal doses (200 ng/mg larva) of stable isotope labelled 3-NPA ([^15^N, 1-^13^C] 3-NPA) were injected into the larval hemolymph of *P. cochleariae*, *C. populi*, and willow-feeding *C. lapponica*. HPLC-MS analyses revealed the incorporation of 3-NPA into isoxazolin-5-one glucoside **5**, forming the corresponding ester **6**. Diesters or triesters, as reported in adult leaf beetle secretions (Matsuda and Sugawara 1980), were absent. Furthermore, the enrichment of the isotope signals at [M + 2] in the hemolymph compared to buffer-treated control groups was determined. The values ranged between 7 and 24.5 %, indicating a tolerance to free 3-NPA (Fig. S5). The isotope enrichment for compound **6** in *C. populi* was 13.2 % ± 4.3 % (arithmetic mean ± standard deviation, *N* = 4 for each species), in the case of *P. cochleariae* it was 7 % ± 1.3 %, while *C. lapponica* showed 24.5 % ± 9.5 % enrichment. In summary, 3-NPA is esterified to **6** with differences in efficiency depending on the examined species. Compound **5** apparently serves as a carrier to attach free 3-NPA to form the non-toxic ester **6**.

### Detection of Salicin and 8-Hydroxygeraniol-8-O-ß-D-glucoside in the Larval Hemolymph

In addition to examining the hemolymph production of isoxazolin-5-one derivatives, we screened for *de novo*-produced as well as sequestered precursor glucosides of the volatile deterrents in the hemolymph (Fig. 1). Under the chosen chromatographic conditions, our target compounds showed signals at *m/z* = 377 [M+HCOO]^−^ for 8-hydroxygeraniol glucoside **3** in *P. cochleariae* as well as *m/z* = 331 [M+HCOO]^−^ for salicin **1** in *C. populi*. The compounds were identified by comparison of HPLC-MS chromatograms of the natural samples with the spectra of commercially available standards. To confirm our identification of previously mentioned glucosides in the hemolymph of *P. cochleariae* and *C. populi,* we reanalyzed the *dqf*-COSY spectra. These confirmed the presence of small amounts of 8-hydroxygeraniol glucoside **3** as well as salicin **1** (Fig. S6). In summary, we identified 8-hydroxygeraniol glucoside **3** and salicin **1** in the hemolymph of *P. cochleariae* and *C. populi*, respectively. This confirms a function of the hemolymph as a transport matrix for the isoxazoline-glucoside **5**, its 3-NPA ester **6,** and the deterrent precursors, produced *de novo* or sequestered, *en route* to the tissue of destination (Discher *et al.*^2009^).

## Discussion

The toxicity of insects often is linked to warning signals (Pasteels *et al.*1983a). The adults of many Chrysomelina species, for example, have aposematic red elytra advertising the toxicity of 3-NPA esters of isoxazolinone glucosides and their break down product 3-NPA. Compared to adults, the larvae possess a strikingly different defense mechanism. When disturbed, they display large droplets that contain secretions from eighteen glands; these droplets change the larvae’s appearance dramatically. As predators often are conservative when assessing the size of their prey, this behavior alone may prevent life-threatening attacks (Cohen *et al.*^1993^). In addition to the appearance of the larvae, their odor also changes since the droplets contain volatile chemicals in high amounts, such as iridoids or salicylaldehyde. Besides their repellent effect on predators, these irritants have nonspecific toxic effects. Iridoids, for example, can bind proteins covalently that might have adverse effects upon ingestion (Kim *et al.*^2000^), whereas salicylaldehyde exhibits a non-specific cytotoxic effects to insect cell cultures (Gross *et al.*^2002^).

**Fig. 4:**
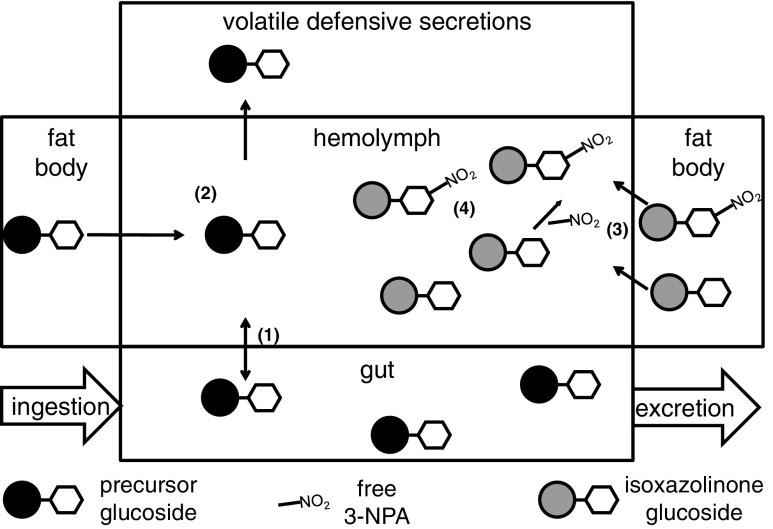
Scheme of glucoside transport in leaf beetle larvae. Glucosides implicated in the volatile defense are ingested with the food. Transport proteins mediate the uptake of glucosides into the hemolymph (**1)**. Precursor glucosides, either sequestered from food or synthesized autogenously, are selectively transported to the defensive glands for further processing **(2)**. Isoxazolinone glucosides are produced in the fat body and released into the hemolymph **(3)**. Free 3-NPA can be conjugated to isoxazolin-5-one glucoside to prevent autointoxication **(4)**

Our discovery of isoxazolinone-based hemolymph toxins led us to revise the view of the defense of Chrysomelina leaf beetles (Fig. 4). The 3-NPA ester **6** itself is a deterrent, as demonstrated with ants (Pasteels *et al.*^1986^; Sugeno and Matsuda 2002). Furthermore, 3-NPA is a cytotoxin that interferes with mitochondrial respiration (Huang *et al.*^2006^). Although adults possess esterase activity in their secretions, and thus, are able to cleave the esters of isoxazolin-5-one glucosides **5** to liberate 3-NPA, it is conceivable that the larvae have to be ingested to release toxic components by predator digestion.

The two described mechanisms of chemical defense, volatile and non-volatile compounds, could have synergistic effects. The odorant signal, *e.g.*, salicylaldehyde, could be a conditioning stimulus, linking the conspicuous odor to the hemolymph toxin. This system, known as olfactory aposematism (Weldon 2013), is effective mainly for vertebrate predators, such as birds; it is how they learn to avoid certain food. As tree-living *Chrysomela* species share their habitat with birds, the strongly odoriferous salicylaldehyde could be especially effective against this category of predators (Topp 1997). Based on the different allomones developed by Chrysomelina beetles, this taxon represents an unrivalled case study in chemical ecology, which illuminates the concerted action of diverse defense strategies during the adaptation of herbivorous insects to a given niche in an ecosystem.

Research into these hemolymph toxins extends our understanding of the chemical defense of chrysomeline leaf beetles considerably (Fig. 3). Sequestering leaf beetle larvae have adapted to use plant-derived precursors to produce their defensive secretions, which has economic advantages but at the same time restricts host-plant affiliation. One Chrysomelina lineage, however, must have escaped the host-linked constraints (precursor uptake) by shifting host-plant families. Consequences for the changing composition of the secretions, for example, were seen in the different populations of the species *C. lapponica*, many of which shifted from salicin-rich willow species to salicin-poor or even salicin-devoid birch species. With the isoxazolinone-based defense, it becomes clear that the larvae are not as dependent on their volatile defense as has been previously suggested. Instead, the hemolymph toxins provide protection, independent from changes in the composition of the repellent secretions after a host-plant shift of a sequestering species such as *C. lapponica.*

Having detected isoaxazolinone glucoside **5** and its 3-NPA esters **6** in *C. lapponica*, *C. populi*, and *P. cochleariae*, we screened additional species of the Chrysomelidae family (see Fig. 3) for isoxazolinone derivatives in the larval hemolymph to obtain an estimate of the occurrence of these defensive compounds. Interestingly, although isoxazolinone glucoside **5** has been found in all analyzed members of the subtribe Chrysomelina, it has been detected neither in the larval hemolymph nor in adult secretions of species of the neighbouring subtribe Chrysolinina or in *Agelastica alni*, a member of the subfamily Galerucinae.

Consequently, the defense based on isoxazolinone derivatives throughout all developmental stages represents a trait unique to Chrysomelina beetles, and as such may be regarded as a chemomarker for this subtribe. For example, *G. depressa*, lately classified as a member of the subtribe Chrysomelina (Pasteels *et al.*^2003^), also contains isoxazolinone glucoside **5**, which supports its classification into this taxon.

Considering the high concentrations of isoxazolinone glucosides **5** and **6** in the larval hemolymph, we asked whether juvenile Chrysomelina beetles derive these compounds from the eggs as a parental gift (Pasteels *et al.*^1986^) or produce them *de novo* during larval development, as has been suggested for adult chrysomelids (Randoux *et al.*^1991^). While an increase in ester compounds **6** during larval development was measured, the concentrations of the isoxazolinone glucoside **5** remained constant (Fig. S4). This suggests that these compounds are produced autogenously during larval development. As none of the respective host plants produce isoxazolinone derivatives, the most plausible scenario for their existence is autogenous synthesis. Furthermore, the increased overall concentration of isoxazolinone derivatives in the hemolymph can result from its lack of excretion by the malpighian tubules, as indicated by the complete absence of **5** and **6** in the larval frass of *P. cochleariae*, *C. populi*, and *C. lapponica*.

Neither compound is exported to the defensive system that encoloses the isoazolinone glucosides **5** and **6** efficiently in the hemolymph. The biosynthesis of the isoxazolinone derivatives most likely starts from the metabolism of amino acids (Randoux *et al.*^1991^), in particular β-alanine, which is efficiently incorporated into **6** (unpublished); however, neither the enzymatic steps of the pathway nor the regulation of the observed ratio of compound **5** to **6** have to date been resolved in the chrysomelids.

Autolysis of isoxazolinone glucoside ester **6** would lead to free 3-NPA and consequently to autointoxication. To address this possibility, stable isotope labelled 3-NPA was injected into Chrysomelina larvae. Labelled isoxazolinone glucoside ester **6** indicated the conjugation of free 3-NPA to the isoxazolinone glucoside **5**. No other detoxification strategies have been found in the Chrysomelina beetles. Such strategies have been reported from other organisms, including the oxidation of 3-NPA that was reported from microbes and plants (Francis *et al.*^2013^), the conjugation to amino acids reported from *Spodoptora littoralis* (Novoselov *et al.*^2015^) and melanopline grasshoppers (Johnson *et al.*^2001^), or the formation of glucosides (miserotoxin) observed in grasshoppers (Johnson *et al.*^2001^). Our findings have two consequences: first, the glucoside and free 3-NPA must be considered as the biosynthetic building blocks of the ester **6**; and second, isoxazolinone glucoside **5** likely serves as a storage site for the neurotoxin, which displays the physiological role of isoxazolinone glucoside **5** in Chrysomelina beetles.

The hemolymph surrounds all organs and is thus a vital transport medium between insect tissues. Intense research over the last decades has demonstrated the existence of a transport network in Chrysomelina larvae that is nonspecific in terms of import of dietary glucosides into the hemolymph (Discher *et al.*^2009^; Strauss *et al.*^2013^). While the larvae excrete glucosides that have not been utilized, they also transport genuine precursors into the defensive glands. In this study, we report for the first time the presence of actual *O*-*β*-d glucosides in the hemolymph of chrysomelid larvae, such as salicin **1** and 8-hydroxygeraniol-8-*O*-*ß*-D-glucoside **3**, which support the transport model presented earlier. Our results lead us to conclude that the open circulation in the hemolymph of Chrysomelina larvae serves on the one hand as a transit site for the glucoside intermediates of the defensive secretions and on the other hand as a storage reservoir for the isoxazolinone derivatives.

## Electronic supplementary material

Below is the link to the electronic supplementary material.

ESM 1 (DOCX 4412 kb)
