# Supplementary material for: Two Defensive Lines in Juvenile Leaf Beetles; Esters of 3-nitropropionic Acid in the Hemolymph and Aposematic Warning
Source: J Chem Ecol. 2016 Mar 31;42:240–8. doi: 10.1007/s10886-016-0684-0 (PMC4839037; doi:10.1007/s10886-016-0684-0)
Supplement: Supplementary file 1 — (DOCX 4412 kb) [file 10886_2016_684_MOESM1_ESM.docx]

Two defensive lines in juvenile leaf beetles; esters of 3-nitropropionic acid in the hemolymph and aposematic warning

*Authors and affiliations*

Gerhard Pauls^1^, Tobias Becker^1^, Peter Rahfeld^1^, Rene R. Gretscher^1^, Christian Paetz^2^, Jacques Pasteels^3^, Stephan H. von. Reuss^1^, Antje Burse^1^, Wilhelm Boland^1*^

^1^ *Department of Bioorganic Chemistry, Max Planck Institute for Chemical Ecology, Jena, Germany*

^2^ *Biosynthesis and Nuclear Magnetic Resonance Group, Max Planck Institute for Chemical Ecology, Jena, Germany*

^3^ *Université Libre de Bruxelles, Department of Biology*

^*^ Corresponding author: Phone: ++49(0)3641-571200, Fax: ++49(0)3641-571202, E-mail: boland@ice.mpg.de

For submission in the **Journal of Chemical Ecology**

**

**Fig. S1** Mass spectra of isoxazoline-5-one-glucoside and isoxazoline-5-one-glucoside ester. Marked signals correspond to [M]^-^ and[M+HCOOH]^-^ ions

**Fig. S2** Mass spectra of isoxazoline-5-one-glucoside ester before and after injection with [^13^C,^15^N]-3-NPA.

The signal intensity of *m/z* 395 corresponding to [M+HCOOH+2] ^-^ is increased, see Fig. S5 for labelling ratios of the investigated species


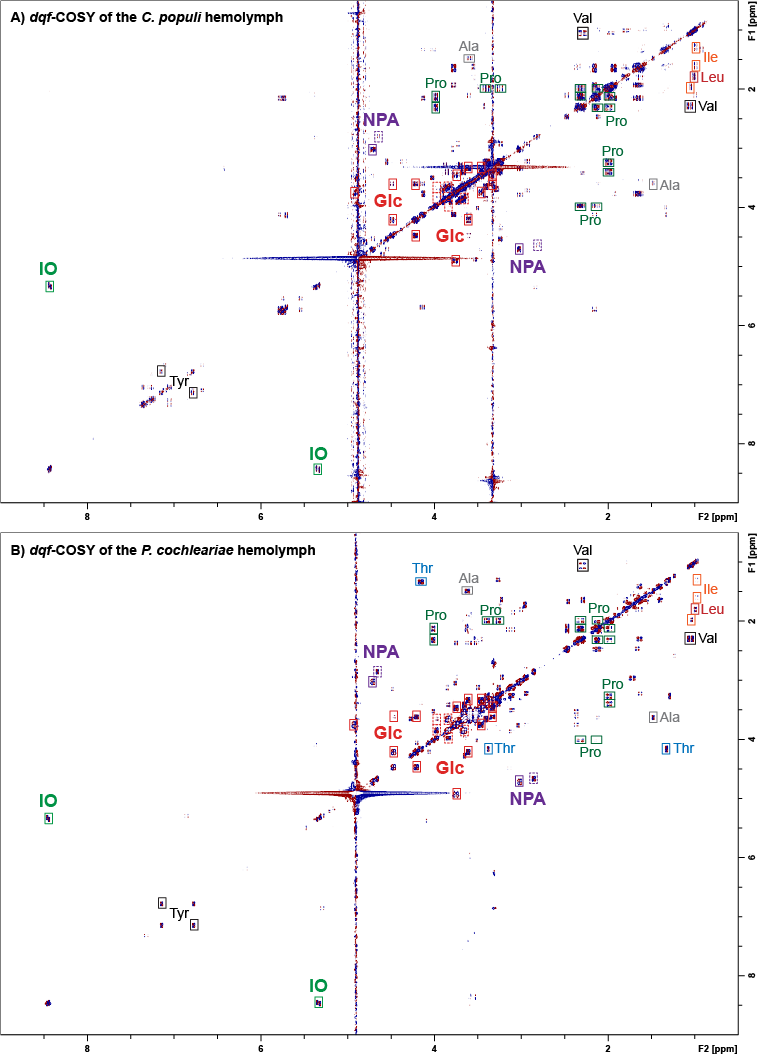


**Fig. S3** Double quantum filtered (*dqf*)-COSY spectra (500 MHz, CD_3_OD) of the crude hemolymph from *Chrysomela* *populi* (**A**) or *Phaedon cochleariae* (**B**). Signals corresponding to amino acids (three letter codes), as well as isoxazoline (IO), glucose (Glc), and 3-nitropropionate (NPA) units are labelled.

**Fig. S4** Concentrations of isoxazolinone glucoside **5** and its 3-Nitropropionic acid ester **6** per mg body weight. The concentration of isoxazolinone glucoside (triangles) remained stable during development (regression analysis, *P*=0.448, *F*=0.593, *N*=30), the ester (dots) showed an increase in concentration (*P*<0.001, *F*=14.757, *r²*= 0.345, *N*=30), indicating autogenous synthesis

**Fig. S5** [^13^C,^15^N]-enrichment of isoxazolin glucoside ester **6** upon injection with [^13^C,^15^N]-3-Nitropropionic acid. The enrichment of **6** upon incorporation of injected labelled 3-NPA was calculated based on the relative intensities of the isotopic signals (detected as formic acid adducts) by using the equation: [^13^C,^15^N]-**6** in % = 100 / ([M+HCOO]^-^ L + [M+1+HCOO]^-^ L + [M+2+HCOO]^-^ L) * ([M+2+HCOO] ^-^ L - [M+2+HCOO] ^-^ C) with L representing the labelled compound and C the unlabelled control. The isotope enrichment for compound **6** in *Chrysomela populi* was 13.2 % ± 4,3% (mean value ± standard deviation, n=10 for each species), in the case of *Phaedon cochleariae* it was 7% ± 1,3%, while *Chrysomela lapponica* showed 24.5% ± 9,5% enrichment.


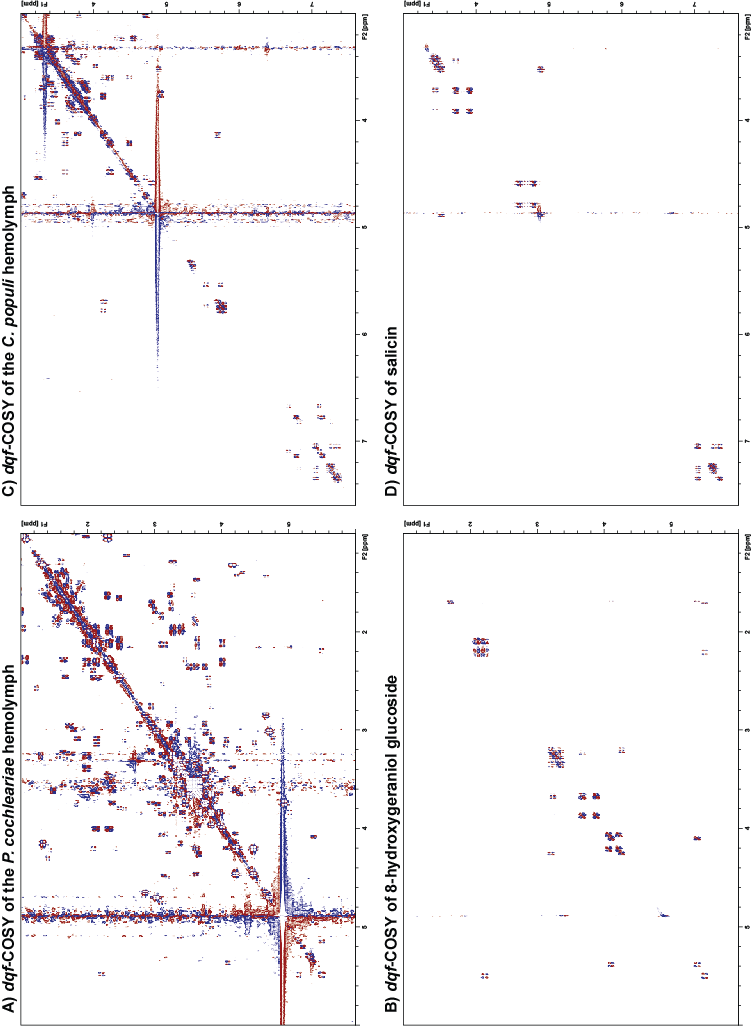


**Fig. S6** NMR spectroscopic identification of 8-hydroxygeraniol glucoside and salicin. **A**: Partial *dqf*-COSY spectrum of the crude *Phaedon* *cochleariae* hemolymph; **B**: *dqf*-COSY spectrum of 8-hydroxygeraniol glucoside standard; **C**: Partial *dqf*-COSY spectrum of the crude *Chrysomela* *populi* hemolymph; **D**: *dqf*-COSY spectrum of salicin standard.


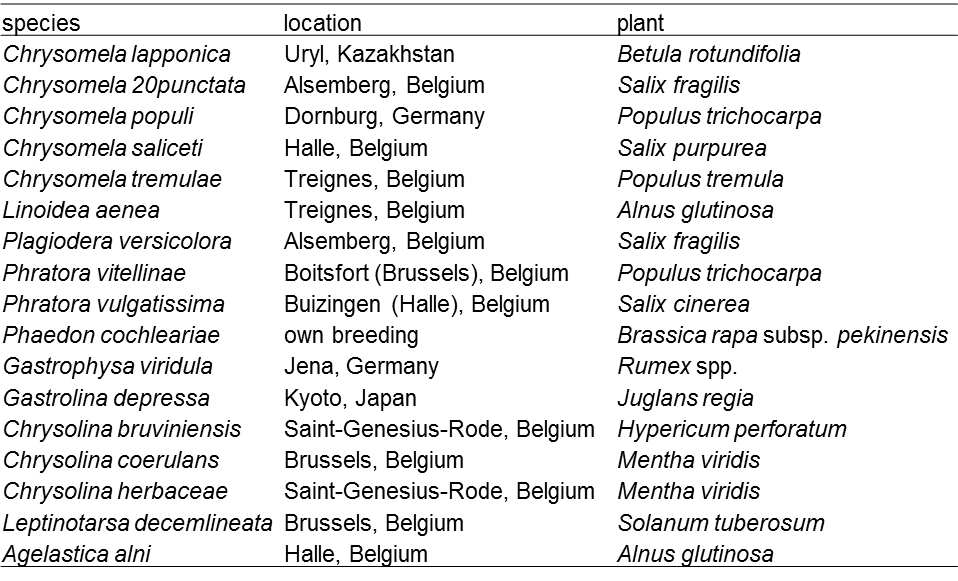


**Tab. S1** Leaf beetle larvae analyzed for this study with collection site and host plant

|  | **5** | | | | **6** | | | |
| --- | --- | --- | --- | --- | --- | --- | --- | --- |
|  | **^1^H** | **COSY** | **^13^C** | **HMBC** | **^1^H** | **COSY** | **^13^C** | **HMBC** |
| 1 | 4.925 d | *J_1,2_* = 9.2 | 90.4* | C-1’,  C-2,3,5 | 4.930 d | *J_1,2_* = 9.2 | 90.2* | C-1’  C-2,3,5 |
| 2 | 3.746* | H-1’, H-3’ | 71.0* | C-1,3 | 3.746* | H-1’, H-3’ | 71.0* | C-1,3 |
| 3 | 3.457* | H-2’, H-4’ | 78.5* | C-2,4 | 3.457* | H-2’, H-4’ | 78.5* | C-2,4 |
| 4 | 3.326* | H-3’, H-5’ | 70.9 | C-3,5,6 | 3.326* | H-3’, H-5’ | 70.9 | C-3,5,6 |
| 5 | 3.407 ddd | *J_5,4_* = 10.8  *J_5,6_* = 5.5 | 80.3 | C-1,3,4 | 3.610 ddd | *J_5,4_* = 10.7  *J_5,6_* = 5.7 | 77.2 | C-1,4 |
| 6a | 3.84 dd 12.2, 2.1 | *^2^J* = 12.2  *J_6,5_* = 2.1 | 62.3 | C-4,5 | 4.466 dd 12.0, 1.9 | *^2^J* = 12.0  *J_6,5_* = 1.9 | 64.7 | C-1’’,  C-4,5 |
| 6b | 3.65 dd | *^2^J* = 12.3  *J_6,5_* = 6.0 |  | C-4,5 | 4.203 dd | *^2^J* = 12.0  *J_6,5_* = 6.0 |  | C-1’’,  C-4,5 |
| 1’ | 8.453 d | *J_1,2_* = 3.7 | 154.8 | C-2’, C-3’ | 8.433 d | *J_1,2_* = 3.7 | 155.1 | C-2’, C-3’ |
| 2’ | 5.315 d | *J_2,1_* = 3.7 | 90.9 | C-1’, C-3’ | 5.344 d | *J_2,1_* = 3.7 | 91.5 | C-1’, C-3’ |
| 3’ | - | - | 174.0 |  | - | - | 173.9 |  |
| 1’’ | - | - | - |  | - | - | 171.5 |  |
| 2’’ | - | - | - |  | 3.016 t | *J* = 5.9 | 31.7 |  |
| 3’’ | - | - | - |  | 4.705 t | *J* = 5.8 | 70.7 | C-1’’,  C-2’’ |

**Tab. S2** NMR data of isoxazolinone glucosides **5** and **6** derived from analysis of ^1^H NMR, *dqf*-COSY, HSQC, and HMBC spectra of *Phaedon cochleariae* hemolymph

**
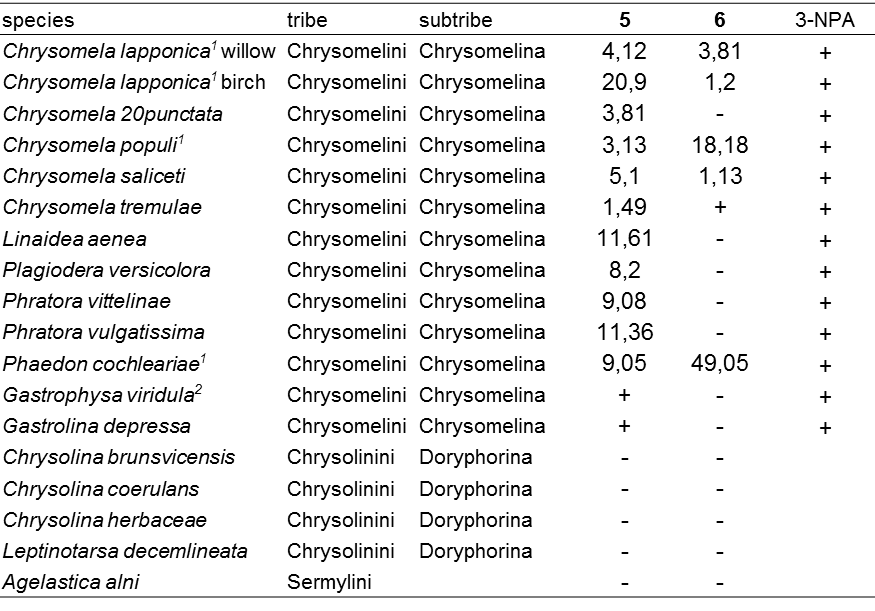
**

**Tab. S3** Screening of larval hemolymph for isoxazolinone glucoside, the corresponding ester and the presence of free 3-Nitropropionic acid. Hemolymph was taken in capillaries, sealed and shipped at RT. ^1^ hemolymph analyzed freshly. ^2^ amounts below limit of quantification. Concentrations are given in nmol/mg hemolymph
